# Seeing with two eyes: How professionals can help patients trying to integrate medical systems

**DOI:** 10.4103/0975-9476.72613

**Published:** 2010

**Authors:** A. V. Balasubramanian

**Affiliations:** *Center for Indian Knowledge Systems, Chennai, India*

## BACKGROUND

Globally today, there is great awareness about complementary health systems[1] and the atmosphere regarding the integration of different medical systems is favorable. Several factors have emerged during the last 20 years that have led to the current situation:

- Transition from an era dominated by communicable diseases to one dominated by systemic diseases and lifestyle disorders
- Greater openness of all medical systems to spell out their limitations, particularly modern biomedical systems, from which powerful criticisms have emerged, yielding better understanding of its limitations
- Greater political support for traditional medicine – recognition by international bodies like WHO of a potentially critical role for traditional medicine[1] in primary healthcare
- Research on history, sociology, and epistemology of medicine leading to better understanding of how CAM systems like Ayurveda are knowledge systems in their own right

Many of these developments have originated in research, either using the methodology of modern western science or with slightly greater openness utilizing, or working toward, interdisciplinary methodologies. This article draws attention to another extremely significant development toward integration of solutions from various medical systems taking place at the level of the patient.

## INTERACTION AT THE GROUND LEVEL

In 1982, after enrolling as a student in the Krishnamacharya Yoga Mandiram (KYM) in Madras (Chennai) as a student of Yoga,[2] I also started teaching Yoga.* My association with the institution has continued over the years even after ceasing to be an active teacher, but more in academic roles. I first became acquainted with the phenomenon of integration of medical systems attempted by the patient, through my observations and discussions both with students and teachers at the Yoga Mandiram and other medical professionals.

Several patients I knew were involved in attempts to integrate inputs and solutions from different medical systems in order to take care of immediate health problems. The systems included modern medicine, Ayurveda, Siddha, Yoga, Naturopathy, and Homeopathy. Also in the scene or background were astrology and religious practices. Generally, one system was chosen for the primary line of intervention, with one or more further systems supplementing it. Choice of primary and supplementary systems was based on a number of factors:

- Exposure to various medical systems based on family background, previous experience
- Extent of exposure to modern/western ideas and
- Nature of the specific condition

Patients seemed to have clear perceptions about the relative strengths of modern medicine and alternative systems. Table 1 summarizes what emerged as the general approach of most patients.

Two illustrations to help clarify this:

**Table 1:** Modern medicine versus alternative systems – Patients’ perceptions of each system’s strengths

Modern medicine	Alternative systems
Surgery	Chronic
Acute conditions	Organic
Emergency situations	Complex situations affecting many physiological systems
Infectious and communicable diseases	Requiring many areas or systems
Problems in specific areas of the body	Psychosomatic conditions
Purely physical problems Analgesic action	Those with strong emotional components

1. In the case of low back pain, students commonly use Yogic asanas (after seeking advice on specific postures) and breathing as the primary line of treatment. Secondarily, they tended to try medicated oils for relief from aches and pains and for suppleness, either as home remedies, or as advised by Ayurvedic practitioners. Only in cases of severe pain were modern painkillers resorted to.
  *Krishnamacharya Yoga Mandiram in Chennai is founded on the tradition of the legendary Yogi, Sri Krishnamacharya (1888–1989) who transformed the teaching of Yoga in the 20th century. He was Guru to a whole generation of famous teachers including B. K. S. Iyengar and Indira Devi who were responsible for wide propagation of Yoga in the West, and Pattabhi Jois and T. K. V. Desikachar, his son, also active both nationally and internationally. KYM (www.kym.org) conducts training programs and research programs, and also offers Yoga diploma courses besides various specialized training programs.
2. In the case of Asthma, Pranayama including breathing in specified postures as advised by Yoga were taken as the primary treatment. Food restrictions imposed by Ayurvedic physicians as pathyam or as advised, or home remedies based on experience, were added. Only in severe attacks were modern medicine’s bronchodilators used.

## FAVORABLE SITUATION

Such attempts to draw on multiple medical systems, and integrate them, are part of a wider phenomenon. They may be attributed to the following:

- The present coexistence of multiple health systems and traditions
- Social organization of knowledge
- Present mindsets being open to synthesis

In India today, these probably occur with much greater frequency and social acceptance.

(1) A significant feature of the Indian situation today is the coexistence of multiple health traditions. A large number of health traditions are legally sanctioned in the sense of being recognized, regulated, and approved by the government. Besides modern medicine, these include Ayurveda, Yoga Medicine and Naturopathy, Unani, Siddha, and Homeopathy, which also enjoy a strong degree of emotional acceptance. In addition, various other systems like Reiki, Pranic healing, and acupuncture have emerged in the last 20 years with varying degrees of support and recognition.

(2) Social organization of indigenous health traditions.

The Indian subcontinent abounds as it were in a variety and diversity of health traditions. The Ayurvedic and Siddha systems of medicine provide us with perhaps the longest unbroken health tradition with not only a stream of practitioners but also textual and theoretical backing.[3,4] These have made their presence felt even outside India, in other parts of Asia such as Tibet, China, Thailand, Cambodia, and Indonesia.

Most remarkable about the Indian medical tradition, however, is that it prevails at two different levels, namely classical systems and folk systems. By “classical systems,” we mean the codified systems consisting of Ayurveda, Siddha, and Unani, and characterized by institutionally trained practitioners, a body of texts, and highly developed theories informing their practices. The folk traditions (which may be called Lok Parampara) consist of oral traditions passed on from father to son, mother to daughter (or daughter-in-law), or guru to sishya in tens of thousands of our villages throughout the ages. Such folk traditions are rich and diverse, and include several kinds of practitioners, as the following illustrates.

- Home remedies and cures for common ailments
- Hundreds of thousands of folk and tribal practitioners known as Vaidus, Nattu Vaidhyars, Bhagats, etc., who learn orally and treat a variety of ailments
- Over 600,000 Dais (traditional birth attendants) who perform home deliveries
- Folklore on health (e.g., proverbs relating to health)
- Knowledge and beliefs regarding foods – Pathyam and Apathyam, i.e., foods to be preferred or avoided during specific diseases, or conditions such as pregnancy, lactation in mothers, etc.
- Knowledge of diagnostic procedures
- Knowledge of preventive measures
- Knowledge of Ritucharya or adaptation of food and regimen to suit the season
- Yoga and other physical cultural practices for preventive and promotive healthcare
- Special areas such as bone setting, Visha Chikitsa (treatment for poisons), Panchakarma (five purificatory procedures), etc.
- Individuals/families specializing in the treatment of specific diseases, e.g., jaundice, asthma, snake-bite

The relationship between folk and classical traditions is found to be symbiotic. There is a strong commonality of underlying theory and world view expressed at the level of Panchamahabhoota (the theory of composition of matter) and Tridosha (the theory of causation of disease). There is also a striking common ground between the technical terms used by expert practitioners and those known to folk practitioners. Sanskrit technical terms and vocabulary from Ayurveda such as Vata, Pitta, Kapha, Ushna, Sheeta, Laghu, Guru, Guna, Veerya, etc., are also very much part of the knowledge of folk practitioners and households.[5]

Ayurveda’s classical texts statements about folk traditions are of interest. Charaka Samhitha says: “Oushadihi naama roopabhyaam, jananthe hyajapaa vane, avipaashchaiva gopaashcha ye cha Anye vanavaasinaha” – “the goat herds, shepherds, cowherds, and other forest dwellers know herbal remedies by name and form….” Similarly Susrutha-Samhitha states: “Gopaalasthaapasaa vyaadha ye chaanye Vana charinaha, Moola jaathihi cha tebhyo Bheshaja vyakthi Ishyathe – “one can learn about herbal medicines from cowherds, tapasvis, hunters, those who live in the forest, and those who live by eating roots and tubers.”

This points to a very important feature of our science and technology namely that its knowledge, theories, and principles should not be the domain of a small number of experts, institutions, or texts, but should be created and shared on a wide scale, even with those ordinary folk who are the day-to-day practitioners of the art of medicine. In fact, though we have used the term “folk knowledge” to denote knowledge with our people for want of a better term, its connotation is quite different in the modern context. The modern Western view uses “folklore” to denote knowledge of supposedly dubious value prevailing among common people and propagated orally, in contrast to “proper” scientific knowledge with its own terminology, theories, and abstractions, which resides in a different body of people viz. experts.

In the Indian tradition, this kind of a sharp qualitative distinction does not seem to exist. “Folk” practitioners are equally innovators at the frontiers of their discipline, and theories and technical categories belong to their domain as well. If we consider, for example, a highly developed branch of Indian Science such as medicine, the basic theories at its foundation, such as the panchabhuta theory of matter and the tridosa theory of causation of disease and its treatment, are common knowledge among the people. Similarly technical terms such as those mentioned above are part of common vocabulary. Here, the expert or specialist plays a different role, namely one of systematizing the corpus of knowledge.

For example, Panini’s grammar, the Ashtadhyayi, is always presented as a paradigmatic example of a perfect theory. Patanjali in his celebrated Mahabhashyam commentary on the Ashtadhyayi discusses theories in detail – what constitutes a theory and how theories are made. For example, in a discussion about the role of the Grammarian, Patanjali says[6]

“He who needs to use a pot goes to a potter’s house and says ‘make me a pot; I need to use one.’ But no one similarly goes to a Grammarian and says ‘Coin words, I shall make use of them.’ He thinks of objects and makes use of words denoting them…the loka (i.e. usage prevailing in the world) is the authority for use of words – ‘Paspasahnika’ of Patanjali Mahabhasyam.”

Thus, there should be no looking down on common folk or lay practitioners. On the contrary, the sastras themselves repeatedly assert that in their concrete particulars and practical use in real-life situations that the truth of the sastras ultimately resides.

(3) Openness to synthesis – a great degree of openness to ideas and solutions from multiple sources and levels seem to be a particular characteristic of the Indian approach. As a principle, Indians seem by and large to have taken the view that meaningful solutions can be accepted and welcome from any source – from the mouth of the child to that of Brihaspati, the divine guru. A well-known verse from the Subhashitam states:

Yuktiyuktam vachograhyam

Baaladapi shukaadapi

Ayuktamapi na grahyam

Saakshadapi brahaspateh

Words that are meaningful may be accepted from any source, be it a child or even a parrot. However, words devoid of reason or meaning should be rejected even if they are originating from Brihaspati, who is honored as the Guru of the Gods.

Even Ayurveda’s classic texts are redolent with this kind of wisdom. The name Charaka, for example, means “wanderer,” with the implication that the Charaka Samhita was a compilation of the experience of vaidya families and folk medical traditions all over India. This openness based on shastric admonition and millennia of experience means that Ayurveda today finds itself in a strong position to integrate (with) other systems of medical knowledge.

## STRENGTHS AND CURRENT EFFORTS AT INTEGRATION

In this light, and as a result of current efforts at integration, we note the following specific strengths in historic Ayurveda, and its contemporary use in integrative practice:

- Practical benefits experienced by the patient
- Modern medicine as a primary line of treatment, with secondary inputs from alternate systems as regards food, improved immunity, and other system capacities, make a fine complementary pairing
- Traditional medicine as the main line of treatment for chronic disease, with modern medicine for acute emergencies, surgical procedures, communicable diseases, etc., also form another fine complementary pairing[7,8]
- Either pairing is now frequently used to take care of several contingencies, e.g., minimizing the side effects of modern cancer treatments[9,10]
- Both have led to mutual respect between practitioners[11]
- Both have paved the way for a better understanding of each others’ strengths
- The patient becomes an active participant in his own healing process

## WEAKNESSES OF THE CURRENT AD HOC EFFORT

While it is significant that efforts at integration are in progress at the ground level, it cannot be denied that they suffer from a number of limitations due to their being ad hoc and without centralized leadership or direction. Some of the weaknesses are as follows:

- One or more experts (sometimes all!) being in the dark about the overall plan! This leads to poor design
- Lack of fall back arrangements for emergencies, when traditional medicine is tried as the main line of treatment
- Practitioners of modern medicine (even open-minded ones) being ill-equipped or unable to give patients advice on complementary therapies – what, where, and how.
- The worldview of many patients still being entrenched in the traditional medicine paradigm which may get projected to the modern treatment with dangerous consequences, e.g., stop taking medicine when symptoms disappear/deep suspicion of long drawn out medication for palliative treatments of chronic conditions
- This also applies in reverse to the modern medicine paradigm which may get projected onto traditional treatments with dangerous consequences, e.g., deep suspicion of slow response to medication for deep treatments of chronic conditions, so stop taking medicine before symptoms are minimized or disappear
- Modern practitioners advising patients on traditional therapies without inputs and expertise of traditional scholars – leading to results that are at best limited and sometimes negative.

As an illustration, consider this example of how modern research on a specific aspect may merely increase confusion when modern practitioners utilize pieces of it ad hoc. A recent review of antidiabetic properties of bittergourd,[12] states in its review of scientific literature “Modern scientific analyses of (bitter gourd’s) antidiabetic properties reveal that it has the capacity to regulate vitiated carbohydrate digestion, glucose metabolism and utilization, possesses insulin mimetic and secretagogue activities, and corrects the impaired antioxidant defence in diabetes.”

Based on this kind of thinking, some modern medical practitioners might suggest that their patients consume bittergourd, or drink its juice, in quantity, based on the underlying thought that “even if it does not help, it can do no harm.” However, Ayurveda cautions against such generalized practice, pointing out that while bittergourd is beneficial to diabetics who are overweight and obese (kapha), it may not have such benefits and could even harm the very different kind of diabetic who is thin and emaciated (vata).[13]

## DANGERS OF “OVERDOSAGE” DUE TO COMBINED USE OF SEVERAL SYSTEMS

In one specific instance which I recall from my days as a Yoga teacher, a student who was on modern allopathic drugs for hypertension came to KYM for treatment. He was given some training in Asanas and breathing and advised that he should keep his doctor informed about it and monitor his blood pressure regularly. One day at home he got up after Yoga practice and felt dizzy and called for help. It was subsequently discovered that the combined effect of the medication and Yoga practice had taken his blood pressure to a very low level – this happened since he did not keep his doctor informed about the Yoga practice that he was doing.

## TRADITIONAL MEDICINE AND SIDE EFFECTS

There is also a common belief that alternative therapies and traditional medicine such as herbal remedies have no side effects and are entirely safe to use. There is certainly a measure of truth in this to the extent that a system of medicine like Ayurveda has methods of assessing and evaluating drug properties. Their standard drugs used in common treatments are indeed free of “side effects,” but only when used by expert practitioners or knowledgeable persons. But this does not mean that any traditional remedy or approach can be picked up and used by anyone at all with the assurance that “even if it does no good it cannot cause any harm.” Even traditional approaches to healing can cause problems if they are not properly employed. The well-known Yoga text Hathayoga Pradipika warns about improper use of pranayama.[14]

- Ayuktabhyasa yogena sarvaroga samudbhavaha. Yoga improperly practiced can give rise to the incidence of any disease.

## TAKING THE IDEA FORWARD

What we see is that in India, with patients already involved in strong movements on the ground to integrate medical systems, we are in an extremely favorable situation. In order to take this forward to the next level, it is essential to involve the participation of experts from various disciplines. Here are some preliminary thoughts.

A starting point can be for a set of experts from various medical systems, for example, modern medicine, Ayurveda, and Yoga, to consult together and pose the following questions concerning their systems’ approach to specific conditions:

- For what subconditions can completely satisfactory total cures be offered?
- Which conditions can be managed palliatively, and effectively taken care of, even though such solutions may not be totally satisfactory?
- For which conditions can contributions only offer partial treatment, even though this may not include management of the core problem?
- For what conditions can little or nothing be done?
- Supplementary questions include the following:
- For each of the above levels, what side effects may require managing?
- What price will such a treatment involve, both in terms of social and mental costs, as well as financial ones?

To begin with, such a consultative process can be introduced for some selected conditions or diseases. Patients need to be active participants in the process, not merely passive recipients of treatment. Ethical considerations concerning illness and treatment available need to be considered, particularly possible implications of withholding proven treatments.

## FAVORABLE EXTERNAL ATMOSPHERE

The atmosphere worldwide is favorable to inputs and solutions from complementary and alternative systems of medicine. The National Centre for Complementary and Alternative Medicine (NCCAM) at the U.S. National Institutes of Health (N.I.H.) is supporting multiple component programs as follows:[15]

- Supporting research
- Educating the public, patients, practitioners, and researchers on all aspects of CAM
- Nurturing and organizing consensus meetings and producing consensus reports
- Identifying core areas requiring clearer understanding and definition
- Providing information on training and accreditation
- Filling in gaps in policies and procedures

Traditional medical practices are now recognized to have distinctly different approaches to diagnosis and treatment of disease. Investigation of traditional practices must be sensitive to such considerations, particularly choice of possible methodologies to validate them. Several recent developments include adoption of the black box approach, and assessment of entire of intervention packages, rather than investigating individual drugs for specific conditions.[16]

## SEEING WITH TWO EYES

I would like to suggest that in solving medical problems, seeing from the perspective of two different medical systems may be compared to the physiology of “seeing with two eyes.”[17] There turn out to be clear advantages in seeing with two eyes:

- Improved resolution at edges, increased contrast, better ability to read when the print is small or illumination is poor
- Information obtained about depth of field gaining “perspective”
- Information provided by two eyes is thus information of a different logical type

Hence approaches using more than one medical system have interesting advantages and should be taken forward. However, we may foresee instances where two such approaches may be so different as to be almost incompatible.

Take for example, the comprehension of morning sickness (termed nausea gravidarum) experienced by women in pregnancy. Modern science understands this phenomenon in terms of levels of certain hormones such as estrogen, human chorionic gonadotropin (HCG), or hypoglycemia, etc. The traditional Ayurvedic view is in stark contrast. The pregnant women are termed “Douhrdini,” literally meaning “endowed with two hridayas.” In this context, hridaya is not merely the physiological heart, but a seat of emotion. The pregnant woman is seen as one who has a second center and in the fetus nurtured by her, a second seat of emotion growing within her! This gives Ayurveda a completely different insight into the mental state of a pregnant woman, her likes and dislikes, inexplicable reversals of tastes and preferences, etc. What is significant is that this is not merely a philosophical understanding, but Ayurveda offers various time-tested practical solutions in terms of how the pregnant woman can be helped through her morning sickness.[18] In such cases the gap between the two approaches is so large that it is not feasible to “integrate” them in any simple manner – but these would be rich areas for research that would highlight foundational differences between the two systems.

## THE WAY FORWARD

In this scenario the way forward calls for openness and humility and an attitude of wanting to continue to learn from all systems of medicine. In a sense, all systems in their foundation have a certain openness. This needs to be revisited so that the attitude can diffuse to the operational level. I recently revisited the hippocratic oath taken by modern medical students upon graduation – it contains the following interesting paragraph.[19]

“I will not be ashamed to say ‘I know not’, nor will I fail to call in my colleagues when the skills of another are needed for a patient’s recovery.”

I would like to close with a passage from a play by the poet Kalidasa, Malavikagnimitram, which provides a beautiful perspective on the choice between the old and the new.

Puranamityeva na sadhu sarvam

Na capi kavyam navamityavadhyam

Santah pareekshyanyatarath bhajanthe

Moodhah para pratyaya neya buddhihi[20]

Not everything should be accepted as good merely because it is old

Nor should anything to be rejected simply because it is new.

The wise make decisions after examining specific details in each case

Those who get carried away by such general labels and descriptions are fools.
